# Communication about sexuality for adolescents with cerebral palsy and complex communication needs: A scoping review with framework synthesis

**DOI:** 10.1111/dmcn.16479

**Published:** 2025-09-10

**Authors:** Megan Walsh, Susan M. Sawyer, Joanne M. Watson, Amie O'Shea, Georgia Cranko, Chris M. Pacheco, Jacinta Pennacchia, Kate L. M. Anderson

**Affiliations:** ^1^ School of Health and Social Development, Faculty of Health Deakin University Geelong Australia; ^2^ Murdoch Children's Research Institute Parkville Australia; ^3^ Centre for Adolescent Health The Royal Children's Hospital Melbourne Melbourne Australia; ^4^ Department of Paediatrics The University of Melbourne Parkville Australia; ^5^ Institute for Health Transformation, Faculty of Health Deakin University Geelong Australia; ^6^ Manipal University Manipal India; ^7^ Consumer Research Partner Australian Centre for Health, Independence, Economic Participation and Value Enhanced Care for Adults and Young Adults with Cerebral Palsy (CP‐Achieve) Melbourne Australia; ^8^ School of Health and Welfare Jönköping University Jönköping Sweden; ^9^ School of Computing Technologies STEM College, RMIT University Melbourne Australia

## Abstract

**Aim:**

To understand communication about sexuality for adolescents with cerebral palsy (CP) and complex communication needs.

**Method:**

We systematically searched primary research on adolescents aged 10 to 24 years with CP and/or complex communication needs. We coded the primary evidence against themes derived from a theoretical framework analysis. Consumer research partners were involved throughout.

**Results:**

Most of the 16 identified papers described adolescents with CP who could speak. While these adolescents engaged in some discussions with peers about sexuality, they also reported an absence of desired communication with peers and health professionals. The evidence about adolescents with complex communication needs centred on communication with teachers and parents or carers, and on vulnerability to abuse and socially appropriate masturbatory behaviours, rather than positive aspects of sexuality.

**Interpretation:**

Given the complexity of their disabilities, adolescents with CP and complex communication needs probably require support to understand and express themselves as sexual and gendered beings. Our findings reveal a sexuality evidence base that fails to address the needs of adolescents with CP during this critical life phase, emphasizing the need for more inclusive, communication‐aware sexuality research.

## Graphical abstract

We undertook a scoping review to understand communication about sexuality for adolescents with cerebral palsy and complex communication needs. We can understand communication about sexuality by examining the content, context, and influencing factors of that communication.

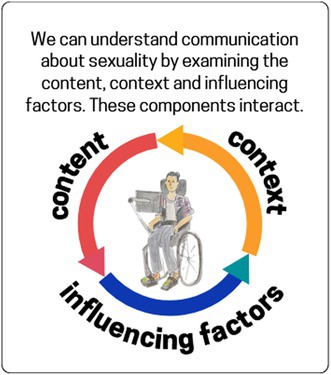

**This scoping review is commented on by Detandt on pages** 8–9 **of this issue.**

Plain language summary: https://onlinelibrary.wiley.com/doi/10.1111/dmcn.70052

**Abbreviations**
LGBTIQ: lesbian, gay, bisexual, transgender, intersex, or questioning
PRISMA: Preferred Reporting Items for Systematic reviews and Meta‐Analyses

**What this paper adds**

- Adolescents with cerebral palsy (CP) want to communicate more about sexuality and with a broader range of people.
- Few studies report on communication about sexuality for adolescents with CP.
- Adolescents with complex communication needs have been especially neglected in sexuality research.
- What adolescents with complex communication needs want from conversations about sexuality remains largely unknown.
- Future research should examine adolescent communication about sexuality, including adolescents' priorities.

Puberty marks the onset of rapid physical, psychological, and social changes as adolescents (aged 10–24 years) form their identities and they come to understand themselves as gendered and sexual beings.^1^ Sexuality encompasses ‘sex, gender identities and roles, sexual orientation, eroticism, pleasure, intimacy and reproduction’,^2^ is shaped by interactions with family and peers, and occurs in a complex social–cultural context.^2^, ^3^

Adolescents with disabilities such as cerebral palsy (CP) experience this developmental period differently from their non‐disabled peers. Nearly 20 years ago, a literature review by Wiegerink et al. reported that adolescents with CP faced barriers to engaging in sexual relationships and dating, including low self‐esteem and overprotective parenting.^4^ People with CP participate socially to a lesser extent than their non‐disabled peers.^5^ This is particularly true for people with CP who also have complex communication needs, meaning they are unable to use speech alone to meet their daily functional communication needs.^6^ Communication disabilities are highly prevalent in people with CP, with up to 50% of those with CP having difficulty speaking and as many as a third not using speech to communicate.^7^, ^8^, ^9^ Barriers to social participation for people with complex communication needs include reduced rate of communication, lack of adequate and available vocabulary for communicating socially, inadequate communication partner skills, and negative societal attitudes.^10^, ^11^

Wiegerink et al. and others have drawn a connection between the social lives and the sexual and romantic lives of adolescents with CP,^4^, ^12^, ^13^, ^14^, ^15^, ^16^ but there is little information about how adolescents with CP communicate about sexuality, with literature pertaining to those with complex communication needs even more scarce.^17^ Communicating about sexuality is important because, as noted, interactions with people such as family and peers profoundly shape the development of sexual identity. We use the terms ‘communicate’ or ‘communication’ rather than ‘talking about’ or ‘conversation’ throughout this paper, as an acknowledgement that some adolescents with complex communication needs may not use words or symbols to communicate yet are still able to engage meaningfully with parents, peers, support workers, and romantic or sexual partners through embodied or alternative modes of expression.

The aim of this scoping review was to critically examine existing research on communication about sexuality for adolescents with CP and complex communication needs. Anticipating a limited evidence base, as suggested in the review by Sellwood et al.,^17^ we chose to undertake searches addressing communication about sexuality in two populations: (1) adolescents with CP and/or (2) adolescents with complex communication needs. Our scoping review maps existing evidence and, importantly, interrogates its theoretical and methodological boundaries, with the goal of informing more inclusive, communication‐aware approaches to adolescent sexuality research.

## METHOD

**FIGURE 1:**
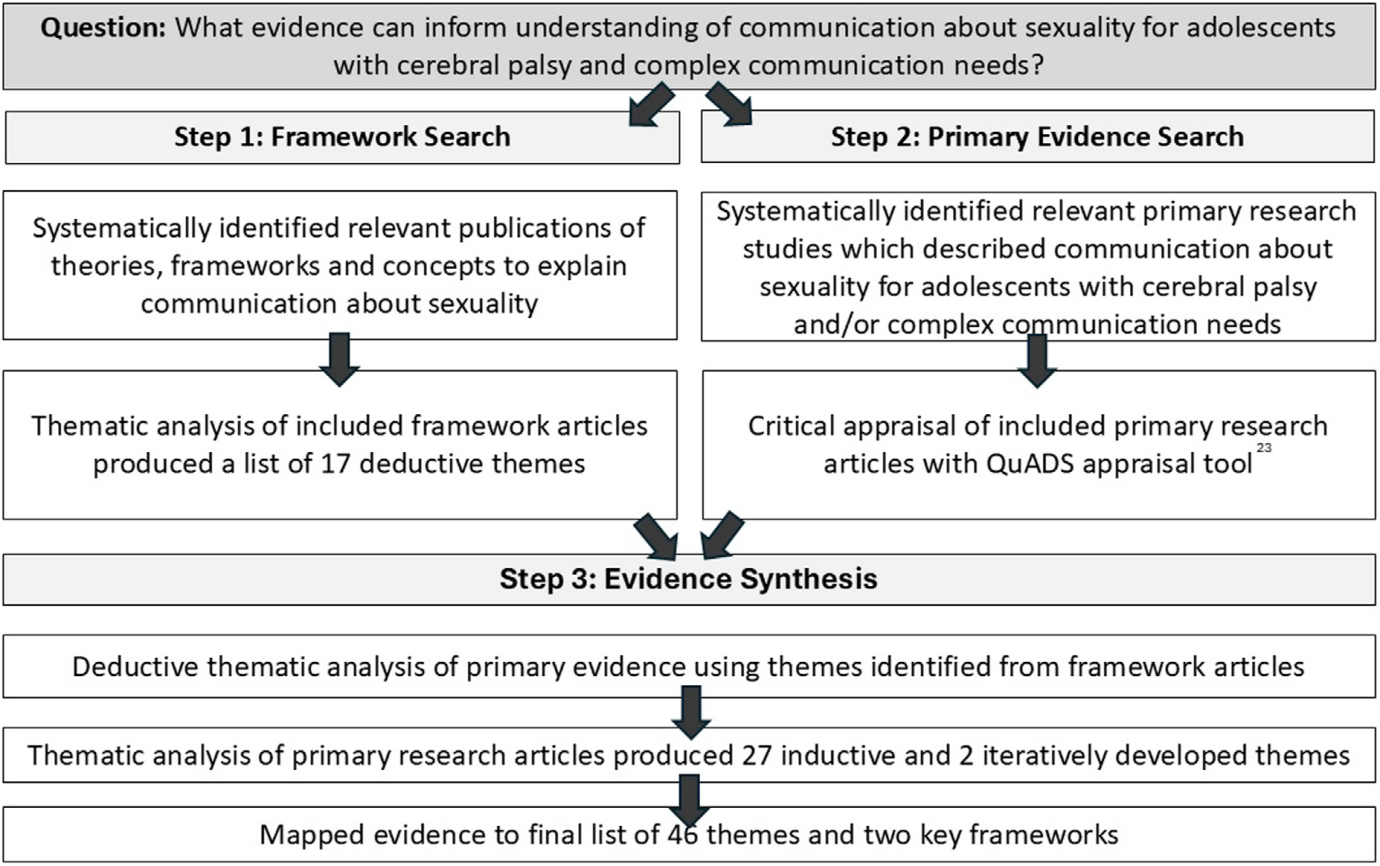
Framework synthesis method.

This review was informed by the ‘best fit’ framework synthesis method of Carroll et al.^18^ and the JBI guidelines for scoping reviews.^19^ The review occurred in three stages (Figure 1). To inform subsequent analysis of the primary research papers, in step 1 we undertook a systematic search of theoretical frameworks relating to communication about sexuality in the general population. This enabled the development of an a priori coding schema. Themes were broad, and included social norms about sexuality, abuse and risk, and digital interactions (e.g. online dating, sexting). In step 2, we sourced and screened primary research relating to interactions about sexuality with adolescents with CP and/or complex communication needs, and critically appraised the included papers. Finally, in step 3 we synthesized the evidence using a double‐coding process. We deductively coded the included papers against the themes produced in step 1 (framework search), and then conducted an inductive thematic analysis of the included papers to identify novel themes. This framework synthesis, or ‘gap‐mapping’ exercise, allowed us to map primary research specific to the sexuality of people with CP and/or complex communication needs and, in doing so, also to highlight key similarities and differences with ‘mainstream’ sexuality research.

For brevity, we have included details about the systematic framework search (step 1) such as the Preferred Reporting Items for Systematic reviews and Meta‐Analyses (PRISMA)^20^, ^21^ flow chart and the list of included papers in the supporting information (Figure S1). The methods and findings from steps 2 and 3 are described below. The study protocol was registered on Open Science Framework (https://osf.io/5c2xt) before data analysis.

### Primary evidence search (step 2)

In June 2023 we completed a systematic search of primary research evidence describing communication about sexuality for adolescents with CP and/or complex communication needs. Searches were completed in EBSCO (Academic Search Complete, APA PsycInfo, CINAHL Complete, Education Source, ERIC, Global Health, LGBTQ+ Source, Medline Complete, and SocINDEX) and Embase, which covered a wide range of disciplines and languages. We searched for key terms at subject heading and full‐text levels using terms associated with sexuality and complex communication needs. The search extended from 1996 to 2023, to capture relevant timepoints in disability (e.g. de‐institutionalization) and sexuality (e.g. HIV/AIDS epidemic) history. The full search strategy for each database is detailed in the published study protocol.

### Primary evidence screening

Papers were selected in line with the PRISMA extension for Scoping Reviews (Figure S2).^20^, ^21^ Using Covidence,^22^ titles and abstracts were independently screened by the first author, with 10% checked by a second reviewer (KA). All full texts were double‐screened by the authorship team. As there are several terms that describe people who have complex communication needs, authors with expertise in speech–language pathology (MW, JP, JW, KA) were responsible for screening papers at the abstract and full‐text levels for the presence of participants with complex communication needs. Studies from any country and written in any language were included in recognition of the significant role that culture plays in the concepts of sexuality and disability. Machine translation was used to translate non‐English titles, abstracts, and/or full texts in the reviewers' primary language (English) for screening. Studies of any case design were included. The inclusion criteria are listed in Table 1, outlining the population, concept, context, and sources of interest for the review. From the primary evidence search, 6150 unique papers were identified. Careful screening and review of these papers against the inclusion criteria resulted in 16 papers that were included in this study (Figure S2).

**TABLE 1:** Inclusion criteria for primary search strategies: population, concept, context, and source criteria for primary evidence.

Population: adolescents with CP and/or complex communication needs	Diagnosis of CP and/or complex communication needs Data for participants with CP and/or complex communication needs can be differentiated from participants with other disabilities Age 10–24 years old when interactions are occurring (if older ages are included, must be able to differentiate data for adolescents) Research participants may be communication partners of adolescents with CP and/or complex communication needs, so long as communication with adolescents is reported (e.g. a parent describing communication about sexuality with their child with CP)
Concept: sexuality	Focus: experiences, views, behaviours, and engagement of target populations about sexuality
	Focus: communication around the concept(s) of sexuality
Context: communication	Must address the involvement of adolescents with CP and/or complex communication needs in these interactions
Source	Peer‐reviewed journal or conference proceedings
	Original, empirical completed research
	Minimum 1000 words
	Any language

Abbreviation: CP, cerebral palsy.

### Critical appraisal of included primary evidence

Given the wide range of study types anticipated, the QuADS appraisal tool^23^ was chosen to present an integrated critique of study quality across disciplines and methods using a summary score (Table 2). To map methodological strengths and weaknesses in the research, we added two additional criteria: whether authors discussed their study's strengths and limitations, and whether they adequately addressed ethical considerations (given the sensitivity of sexuality and disability). Author MW completed appraisal of all papers, with 25% also evaluated by JP; any differences were resolved through discussion.^23^ Rather than simply using the QuADS score to denote high or low quality, researchers are advised to construct a narrative quality analysis based on the scores for each criterion across the included literature,^23^ which we also provide.

**TABLE 2:** Primary research studies.

Study	Population	Informants	Country	Method	Summary of findings related to interactions about sexuality	QuADs appraisal tool^23^ score
Björquist et al.^33^	17‐ to 18‐year‐olds with CP across a range of gross motor function and cognitive abilities	12 adolescents	Sweden	Focus groups, individual interviews	Absence of but desire for romantic partners. Experienced stigma from peers.	33/42
Cummins et al.^24^	11‐ to 19‐year‐old females with autism and intellectual disability who are ‘minimally verbal’	10 parents, 10 educators of 12 females	UK, Ireland	Semi‐structured interviews	Found that parents and educators focused on teaching independence in managing menstruation, prevention of abuse, and managing sexual behaviour.	17/42
Czapla and Otrębski^25^	15‐ to 25‐year‐olds with CP and ‘normal intelligence’	61 adolescents	Poland	Survey	Two out of three respondents had low sexual self‐esteem and low sexual needs.	17/42
Czapla and Otrębski^26^	Same cohort as Czapla and Otrębski^25^	62 adolescents	Poland	Survey	Adolescents were engaged in sexual behaviours; the frequency of engaging in those behaviours was related to levels of sexual self‐esteem and sexual need.	19/42
Davis et al.^34^	13‐ to 18‐year‐olds with CP across a range of GMFCS levels	17 adolescents, 23 parents	Australia	Interviews	Parents identified sexuality and relationships as important facets of quality of life for their children. Adolescents discussed self‐esteem, stigma, and acceptance of disability.	32/42
East and Orchard^35^	15‐ to 20‐year‐olds with a physical disability that requires a mobility device (excluded adolescents with ‘additional cognitive or developmental disabilities’)	Adolescents, parents; data available for 2 adolescents with CP and 1 parent	Canada	Narrative interviews	Adolescents and a parent discussed the experiences and needs of sexual health and education for adolescents with physical disabilities.	32/42
East and Orchard^36^	Same adolescent sample as East and Orchard^45^	Adolescents. Data available for 2 adolescents with CP	Canada	Narrative interviews	Adolescents reported experiences related to stigma, self‐esteem, social participation, and sexual and romantic relationships.	25/42
Power et al.^37^	10‐ to 18‐year‐old young females with CP across a range of GMFCS levels and a range of associated impairments	12 adolescents, 33 primary female caregivers (including 21 caregivers reporting on adolescents who were unable to self‐report)	Bangladesh	Focus group discussion	Centred on menstruation experiences for adolescent females with CP, highlighting the influence of cultural and social norms in navigating puberty. Data available for adolescents with complex communication needs focused on functional support needs, shame/social norms, and fear of sexual abuse.	35/42
Power et al.^38^	10‐ to 18‐year‐olds with CP across a range of GMFCS levels and with a range of associated impairments	24 adolescents, 76 primary caregivers *Note: this sample includes the 12 female participants from Power et al.^37^	Bangladesh	Focus group discussion	Focused on sexual maturity, highlighting wider gender roles and opportunities, but also exclusion from many of those opportunities. Available data for adolescents with complex communication needs focused on functional support needs, shame/social norms, and fear of sexual abuse.	34/42
Qian^27^	3‐ to 17‐year‐olds with disabilities	6 orphanage‐based caregivers and extractable data available for 5 adolescents with CP, including 1 with complex communication needs	China	Embedded ethnography. Formal and informal interviews with orphanage‐based caregivers; observation of adolescents	Sexuality in children and adolescents with disabilities viewed as deviant and dangerous by the orphanage‐based caregivers (nannies).	17/42
Shikako‐Thomas et al.^39^	12‐ to 19‐year‐olds with CP across a range of GMFCS levels, with a range of cognitive abilities	14 parents of 16 adolescents	Canada	Interviews	Parents talked to their adolescents about masturbation, dating, and sex. Parents were thinking about social opportunities, independence, and risk of abuse for their adolescents.	33/42
Wiegerink* et al.^16^	T1: 16‐ to 20‐year‐olds	T1: 103 adolescents	the Netherlands	Structured interviews, surveys	Described the ‘social, intimate and sexual relationships’ of the sample with CP compared with able‐bodied age‐matched peers. Reported that 30% functioned socially below their peers, and many struggled to establish romantic relationships.	30/42
Wiegerink* et al.^14^	Prospective longitudinal, 3 time points: 16‐ to 24‐year‐olds	T1, T2 and T3: 74 adolescents (at 4‐year follow‐up measurement)	the Netherlands	Structured interview, survey	Longitudinal study that described adolescents' dating experiences, romantic relationships, and sexual intercourse compared with able‐bodied peers, finding that those with CP participated ‘at a lower level in romantic relationships and sexual activities’.	28/42
Wiegerink* et al.^13^	T2: 18‐to 22‐year‐olds	T2: 87 adolescents	the Netherlands	Structured interviews, surveys	Described romantic relationships with a particular focus on social participation. Greater engagement in peer group activities was linked to development of romantic relationships and engagement in sexual activity.	27/42
Wiegerink* et al.^12^	Prospective longitudinal, 3 time points: 16‐ to 24‐year‐olds	T1, T2, and T3: 74 adolescents (at T3)	the Netherlands	Surveys	Described the sexual experience, sexual problems, and sexual information needs of the cohort over time.	19/42
Wiegerink* et al.^15^	T3: 20‐ to 25‐year‐olds with CP	T3: 74 adolescents (at 4‐year follow‐up measurement)	the Netherlands	Structured interviews, surveys	Explored personal, environmental, and physical factors associated with sexual and romantic activity at T3, with a positive association between self‐esteem and self‐efficacy and being in a romantic relationship.	26/42

Abbreviations: CP, cerebral palsy; GMFCS, Gross Motor Function Classification System; T1, T2, T3, baseline, follow‐up at 2 years, follow‐up at 4 years.

We have reported findings from low‐quality studies with caution, noting methodological limitations. For example, the study by Cummins et al.^24^ was rated as moderate quality. However, because it was the only article exclusively about adolescents with complex communication needs, it received considerable attention in the review. A second example was the two papers by Czapla and Otrębski,^25^, ^26^ which were rated as poor quality; we reported on themes from their work, but only when those themes also appeared in other included papers (in line with what Carroll et al. describe as ‘qualitative sensitivity analysis’^18^). Finally, we found that the QuADS was not the most appropriate tool for appraising the embedded ethnographic method of Qian.^27^ Upon closer examination, we found that Qian's work exhibited stronger methodological rigor than its QuADS score suggested, highlighting a limitation in the tool's applicability to embedded ethnographic research.

### Evidence synthesis (step 3)

**FIGURE 2:**
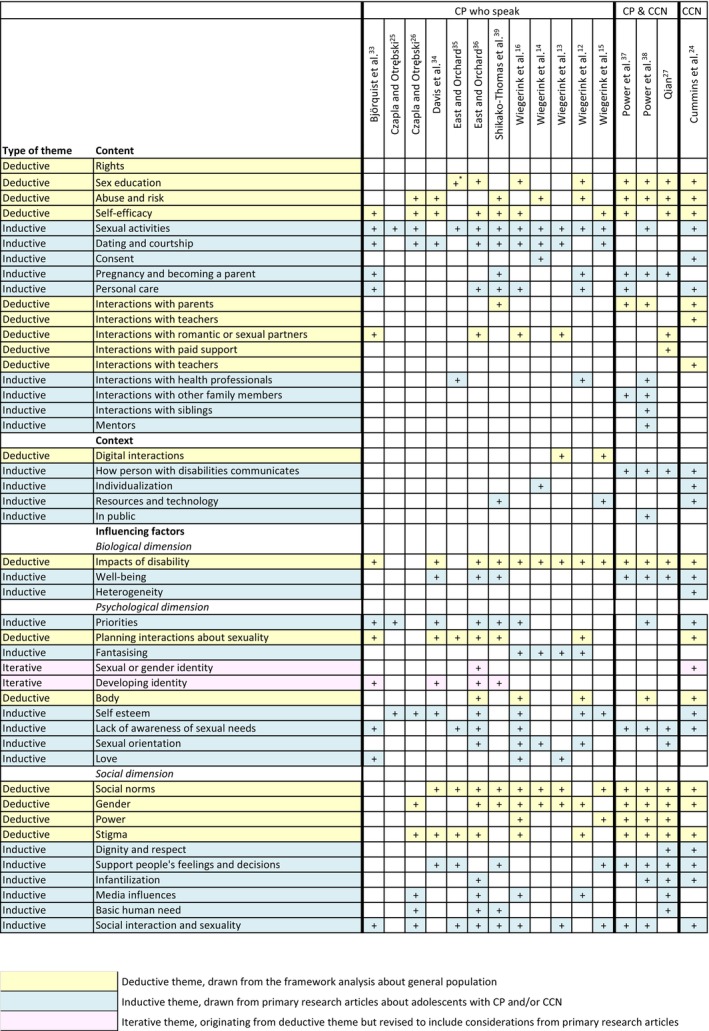
Themes from thematic analysis, mapped onto primary research papers (step 3). + indicates that the theme appeared at least once in the article. Abbreviations: CP, cerebral palsy; CCN, complex communication needs.

Evidence synthesis followed the analysis approach outlined by Carroll et al.^18^ Evidence was first coded deductively against 17 themes identified in step 1 (framework search) and then coded inductively to produce additional themes if appropriate/warranted. The complete list of themes (Figure 2) was organized into three overarching categories: (1) content (e.g. what the interactions are about and with whom); (2) context (mechanics of conversations, support required, and location); the and (3) influencing factors (e.g. impact of disability, stigma, social participation). Figure 2 also illustrates the distribution of these themes across the primary research papers and target population groups. During the evidence synthesis, we identified two relevant frameworks (theory of planned behaviour^28^ and biopsychosocial model^29^, ^30^). These frameworks are further explored in the Discussion, particularly in relation to how they help connect themes in the primary literature.

### Consumer involvement in this systematic review

The authors of the Guidance for Reporting Involvement of Patients and the Public 2 checklist recommend that public engagement in health and social care research should be transparently and comprehensively reported.^31^ Author GC is a person with CP and complex communication needs and author CMP is a parent of a child with CP and complex communication needs. Our thematic analysis and discussions were additionally informed by consultations with a broader advisory group consisting of people with CP and complex communication needs, parents, and a person with CP who speaks. Additional information on consumer involvement in this review, including a critical reflection, is in Table S1.

Consumers provided advice on the use of the term ‘complex communication needs’ to describe our target group's communication. Although the advisory group members preferred descriptive terms such as ‘uses a device to communicate,’ they acknowledged and accepted that these narrower definitions did not fit our review's broader scope. However, the advisory group members indicated that the acronym ‘CCN’ is both confusing and feels like yet another label, mirroring findings from the survey of Zisk and Konyn.^32^ Consequently, although in this paper the term ‘complex communication needs’ is used, it is not abbreviated.

## RESULTS

### Characteristics of the sources of evidence: primary research

A summary of the 16 primary research papers is shown in Table 2.

### Participants

Eight novel groups of participants (and one group with some novel and some common participants) were described across the 16 primary papers reviewed. The most notable overlap came from Wiegerink et al.,^12^, ^13^, ^14^, ^15^, ^16^ whose five papers focused on different results (e.g. social participation and romantic relationships, sexual information needs) or time points (e.g. age) for the same cohort of adolescents with CP.

Young people aged 10 to 25 years were represented across the included literature, with one study^27^ also including younger children. Twelve of the 16 papers exclusively addressed CP.^12^, ^13^, ^14^, ^15^, ^16^, ^25^, ^26^, ^33^, ^34^, ^37^, ^38^, ^39^ An additional three papers included broader populations from which data specific to adolescents with CP were extracted.^27^, ^35^, ^36^ Every paper that reported on Gross Motor Function Classification System (GMFCS) level included participants whose CP was classified in all five levels.^12^, ^13^, ^14^, ^15^, ^16^, ^34^, ^37^, ^38^, ^39^ The papers that included adolescent self‐reporting predominantly represented adolescents in GMFCS levels I and II.^12^, ^13^, ^14^, ^15^, ^16^, ^37^, ^38^

Only 4 of the 16 papers addressed communication about sexuality with adolescents with complex communication needs.^24^, ^27^, ^37^, ^38^ Three of those papers contained data about adolescents with complex communication needs which could be extracted from the broader population with CP.^27^, ^37^, ^38^ The remaining paper focused on adolescent females (11–19 years) with complex communication needs in the context of autism and intellectual disability.^24^

All 16 papers reported on sex as a binary variable; none mentioned asking participants about their gender identity, differentiation between gender and sex assigned at birth, nor innate variations of sex characteristics (also known as intersex). Two papers reported the sexual orientation of adolescent participants.^14^, ^16^

### Primary research study methods

Four of the 16 papers used focus groups with adolescents and parents or carers.^33^, ^35^, ^37^, ^38^ Eleven papers consisted of individual interviews with adolescent participants,^13^, ^14^, ^15^, ^16^, ^33^, ^34^, ^35^, ^36^ parents,^24^, ^34^, ^35^, ^39^ educators,^24^ and orphanage carers (nannies).^27^ Seven papers from two cohort studies used written surveys with adolescents with CP who speak.^12^, ^13^, ^14^, ^15^, ^16^, ^25^, ^26^ Seven papers adopted a mixed methods approach.^13^, ^14^, ^15^, ^16^, ^27^, ^33^, ^35^ Qian was the only researcher to include ethnographic observation as part of her methods.^27^ Nine papers used qualitative analyses.^24^, ^27^, ^33^, ^34^, ^35^, ^36^, ^37^, ^38^, ^39^

In contrast to their peers with CP who spoke, adolescents with complex communication needs were not directly involved in data collection. Rather, data involving them captured the perspective of their supporters such as carers, teachers, or parents.^24^, ^27^, ^37^, ^38^ Cummins et al. attempted a bespoke card‐sorting task in an effort to involve adolescent females aged 11 to 19 years with complex communication needs, but did not include any direct findings.^24^

### Synthesis of results

Because the quantitative studies' methods were disparate, we used a descriptive analytical method to synthesize and interpret quantitative results.^40^, ^41^ Below, we qualitatively describe the results of the primary research, organized into three overarching categories: the content of the communication, the context, and influencing factors. We found there were differences between population groups—adolescents with CP who speak and adolescents with complex communication needs—and as such those differences are reported where relevant.

### Content: the ‘what’ of communication about sexuality

Information about the ‘content’ of communication about sexuality was reported in eight papers for people with CP who speak^12^, ^13^, ^16^, ^35^, ^36^, ^37^, ^38^, ^39^ and three papers for people with complex communication needs.^24^, ^37^, ^38^ Some communication topics were common to both adolescents with CP who speak and adolescents with complex communication needs. Participants in both populations had received some sex education which also included content on abuse.^12^, ^13^, ^16^, ^24^, ^35^, ^36^, ^37^, ^38^ Both populations had discussed puberty (including menstruation) with parents and teachers,^12^, ^16^, ^24^, ^35^, ^38^ while adolescents with CP who could speak had also discussed puberty and menstruation with friends and other family members.^37^, ^38^ Communication with parents about masturbation was reported for both populations,^24^, ^38^, ^39^ although for adolescents with complex communication needs, communication about masturbation seemed to have primarily focused on behaviour management.^24^, ^38^ Parents of both populations discussed self‐efficacy with their adolescents in the context of increasing independence (e.g. managing menstruation^24^, ^37^ or dating without a chaperone^39^).

Some topics related to sexuality were specific to one population group. For example, we found evidence that adolescents with CP who speak discussed topics such as marriage with their peers,^37^, ^38^ and dating, sexual activity, gender roles, and becoming a parent with their peers and parents.^13^, ^16^, ^34^, ^36^, ^37^, ^38^, ^39^ Communication about these topics was not reported for adolescents with complex communication needs.

Nine studies identified that adolescents with CP who speak are dating, falling in love, and having sex,^12^, ^13^, ^14^, ^15^, ^16^, ^25^, ^26^, ^33^, ^36^ but none of the included papers described communication between adolescents and their romantic or sexual partners. Adolescents with CP who speak reported the desire for, but absence of, communication about sexuality with romantic or sexual partners^33^, ^36^ and peers.^33^, ^35^, ^37^ They also reported the need for sexual health information specific to their disability^13^, ^16^, ^35^, ^36^, ^37^, ^38^ such as the impact of CP on fertility or how people with CP can have sex.^12^ In two papers, adolescents reported a lack of communication with health professionals about sexual health.^12^, ^35^ There were no comparable data reported for adolescents with complex communication needs.

Communication between adolescents and their support workers was not reported on for either population (beyond the unique orphanage setting reported by Qian in China,^27^ described below), despite the need for assistance with personal care.^12^, ^16^, ^24^, ^33^, ^36^, ^37^, ^39^ Some adolescents with CP who speak reported needing assistance for sex.^12^, ^16^ Personal care was reported as a topic of conversation between adolescents with complex communication needs and their parents or primary carers,^24^, ^37^ but was not reported for adolescents with CP who speak.

### Context: the ‘how’ and ‘where’ of communication about sexuality

Two of the 16 papers described how adolescents with complex communication needs communicated with others, including how they used body language, facial expressions, gestures, and behaviour to communicate about pain and emotions.^24^, ^37^ For adolescents with CP who speak, methods of communicating about sexuality were not reported. Parents of both populations provided supports to their children to prepare them for communicating about sexuality,^24^, ^34^ with teachers also providing supports to adolescents with complex communication needs.^24^ In one study, parents of adolescent females with complex communication needs identified the need for resources to support them to teach their children about aspects of sexuality including menstruation and masturbation.^24^

Specific contexts for interactions about sexuality (e.g. where communication took place) were identified through the deductive and inductive coding process. Contrasting with mainstream work on ‘sexual interaction in digital contexts’^29^ (e.g. sexting, online dating), adolescents with CP who speak briefly mentioned online dating but did not describe online communication.^13^, ^15^ A second context, ‘in public’, came from our inductive analysis and was only described in relation to adolescents with complex communication needs. In the two papers by Power et al., parents and carers reported that they struggled to manage adolescents' socially inappropriate sexual behaviour in public,^37^, ^38^ while in the paper by Cummins et al., parents and teachers focused on teaching adolescents about private and appropriate places to masturbate.^24^

Unique among the papers was the study by Qian, which involved embedded ethnographic research about the role of a nanny in a rural Chinese orphanage that predominantly cared for children and adolescents with disabilities.^27^ Her research documented sexual and intimate encounters, including instances of abuse, between residents of all genders. Qian argued that while she understood that the adolescents were developing sexual awareness, agency, and identity, the nannies believed that adolescents with disabilities could not develop such agency (e.g. were unable to consent). Any signs of sexual development were therefore understood as deviant and dangerous. While some of Qian's findings are uniquely applicable to that institutional environment and culture, other findings substantiated the themes identified in the remaining 15 papers and are included in this review.^27^

### Factors that influence communication about sexuality

As outlined in Figure 2, much of the literature addressed factors influencing communication about sexuality. Guided by the bio‐psycho‐social model—identified in two framework papers^29^, ^30^ and applied during thematic analysis—the following sections examine the biological, psychological, and social factors shaping communication about sexuality.

Eight papers examined the biological influences on communication about sexuality, particularly in relation to adolescents with disabilities.^12^, ^13^, ^14^, ^15^, ^24^, ^35^, ^37^, ^38^ For adolescents with CP, motor limitations affected their ability to engage in masturbation and other forms of sexual activity independently, indicating a need to ask for support (e.g. communicate about) these activities.^12^, ^13^, ^14^, ^15^ Beyond these physical constraints, proxy reports in three papers indicated that for some adolescents with complex communication needs and intellectual disabilities, receptive language difficulties and challenges in understanding social norms further restricted their ability to engage in conversations about sexuality, consent, and interpersonal relationships.^24^, ^37^, ^38^

Several psychological factors influencing how adolescents with CP who speak communicate about sexuality were identified in six papers.^16^, ^25^, ^33^, ^34^, ^36^, ^38^ These factors included their priorities around sexuality, planning interactions about sexuality, self‐esteem, identity, and stigma. Priorities such as sex, love, finding a partner, getting married, and having children shaped how they engaged in conversations about sexuality.^16^, ^25^, ^33^, ^34^, ^36^, ^38^ Additionally, while not directly connected to sexuality, adolescents with CP who speak also emphasized the importance of independence, friendship, and social inclusion.^33^, ^34^, ^38^

Six of the 12 papers that reviewed frameworks focused on how a person imagines, expects, or plans communication around sexuality.^28^, ^29^, ^42^, ^43^, ^44^, ^45^ These six papers each commented that how an individual thinks about communication about sexuality is affected by social norms. Scant evidence existed in the primary research evidence to explain how adolescents with CP imagine or plan for communication about sexuality, such as planning to talk to someone they are romantically interested in, or have a ‘crush’ on. Studies that did address this were limited to adolescents who speak, and centred on how the adolescents balanced their desires, the potential impacts of their disability, and societal attitudes when planning or imagining communication about sexuality.^12^, ^35^, ^36^, ^39^

Another component of the psychological dimension reported was developing identity. Reporting on adolescents with CP who speak, one paper drew a connection between typical adolescent development of sexual and gender identity and the development of an identity as a disabled person.^36^ In adolescents with CP who speak, several psychological components of sexual and gender identity were reported to shape communication about sexuality, including body image,^12^, ^16^, ^36^ self‐esteem,^12^, ^15^, ^16^, ^25^, ^26^, ^33^, ^34^, ^36^ sexual esteem,^15^, ^16^, ^25^, ^26^ and lack of awareness of sexual needs.^15^, ^16^, ^25^, ^26^, ^33^, ^34^, ^36^, ^38^ Wiegerink et al. found that high self‐esteem facilitated flirting and dating.^15^, ^16^ Sexual esteem was defined as ‘a positive regard for and confidence in an individual's capacity to experience their sexuality in a satisfying and enjoyable way’ (Taleporos and McCabe, quoted in Czapla and Otrębski^26^). While elements of the psychological dimension such as priorities, planning, and identity development were identified as influencing factors around communication about sexuality for adolescents with CP who speak, they were not reported for adolescents with complex communication needs.

Stigma was a strong theme and highlighted the interplay between psychological and social dimensions. We coded ‘stigma’ related to sexuality in 11 papers,^12^, ^16^, ^24^, ^26^, ^27^, ^33^, ^34^, ^35^, ^36^, ^37^, ^38^ manifest as infantilization (being viewed as a child, devoid of sexuality and needing protection)^26^, ^27^, ^35^, ^37^ and the idea that physical disability is inherently unattractive.^12^, ^33^, ^34^, ^36^ Adolescents with CP who speak reported internalizing stigma,^16^, ^34^, ^36^ affecting their self‐concept and approach to dating.

Eleven papers about adolescents with CP who speak described a link between communication about sexuality and social factors.^13^, ^15^, ^16^, ^26^, ^33^, ^34^, ^35^, ^36^, ^37^, ^38^, ^39^ This included associating fewer friendships and fewer social opportunities with less dating and fewer romantic relationships. Several factors seemed to contribute to fewer opportunities to communicate about sexuality with peers and sexual or romantic partners, including peers perpetuating stigma,^35^ parental protection leading to a reduction of privacy with friends or potential romantic partners,^38^, ^39^ and the presence of a support person.^12^, ^16^, ^34^, ^39^ Participants in the research of East and Orchard hypothesized that lack of disability content in mainstream sex education^35^ and lack of media representation^36^ prevented peers from communicating with them about sexuality.

A significant focus of the included literature was parents' and carers' attitudes towards sexuality. Parents were informants in six studies,^24^, ^34^, ^35^, ^37^, ^38^, ^39^ particularly in research concerning adolescents with complex communication needs. Following the example of Astle et al.,^28^ we applied the theory of planned behaviour—identified during the framework search (step 1)—to examine how parents' and carers' attitudes, perceived norms, and behavioural control may influence their approach to communication about sexuality with their adolescent. A key parental attitude was concern about their adolescent's vulnerability to abuse.^24^, ^35^, ^37^, ^38^, ^39^ For parents in the study by Cummins et al.,^24^ prevention of abuse was a powerful motivator for teaching adolescent females with complex communication needs how to say ‘no’ to unwanted touch. Out of fear that a support worker would abuse their adolescent, parents of adolescents with CP who speak in the study by Shikako‐Thomas et al. chose to facilitate sexual experiences and provide personal care themselves rather than engage a paid support worker.^39^ While this implies communication about the supports needed for sexual activity and personal care, description of that communication was not reported. Related to subjective norms, parents of adolescents with CP who speak were aware of societal norms around disability and sexuality, and assumed their child would face rejection from potential romantic or sexual partners.^33^, ^34^ Finally, parents of both populations demonstrated the perceived behavioural control that Astle et al. described,^28^ for example by seeking out help from health professionals,^24^, ^37^, ^38^ providing direction to support workers on how to provide supports,^24^ and teaching their adolescent self‐efficacy and communication skills related to sexuality.^24^, ^34^, ^37^

## DISCUSSION

The available evidence concerning how adolescents with CP communicate about sexuality is limited. In this small literature base, we found that adolescents with CP who speak are engaging in romantic relationships and sexual activities, and that they talk about sexuality and relationships with parents, carers, educators, and to some extent peers. Communication spanned a range of sexuality and gender topics including puberty, marriage, becoming a parent, sex, and dating. Adolescents with CP who speak also reported the absence of, but desire for, communication about sexuality with peers, romantic and sexual partners, and health professionals.

This review found remarkably few studies on communication about sexuality for adolescents with complex communication needs, who make up approximately 30% of the population of adolescents with CP.^7^, ^8^, ^9^ The limited evidence we did find suggests that adolescents with complex communication needs and CP interact with fewer people, and discuss fewer topics related to sexuality, compared with those with CP who speak. Communication about sexuality often revolved around abuse, personal care, and managing sexual behaviour—similar to findings in adults with intellectual disabilities.^46^, ^47^

The work of O'Shea and Frawley with females with intellectual disability and that of Wilson with males with intellectual disability highlight the intersecting barriers of societal stigma and ableist attitudes on understanding and navigating sexual identities.^30^, ^46^, ^47^ Extending on this, we propose that intersecting broader social norms and structures, such as stigma and ableism, diminish more positive expressions of sexuality. This has implications for researchers and policy‐makers, who are encouraged to confront infantilization and ableist attitudes in their methodological approaches and policy development. Stakeholders supporting adolescents with CP and complex communication needs—including clinicians, caregivers, parents, and fellow users of augmentative and alternative communication—can combat infantilization by recognizing and validating adolescents' evolving sexual and gender identities.

For both populations, there was limited evidence about where and how communication about sexuality takes place. While our initial framework search of mainstream sexuality literature flagged digital contexts (e.g. online dating, sexting) as being potentially important, only two studies about adolescents with CP reported on online dating,^13^, ^15^ and no other digital contexts were described. Despite experiencing barriers to digital access,^48^, ^49^ adults with congenital physical disabilities and complex communication needs, including adults with CP, have elsewhere reported that online dating gave them an opportunity to choose when to disclose their disability and avoid interference from ableist people in their lives.^50^ More research on the potential role and experiences of online interactions about sexuality for adolescents with CP and complex communication needs is warranted.

We expected that vocabulary availability, communication resources (e.g. use of a communication supporter), and communication mode (e.g. how someone communicates) would be critical factors in understanding communication about sexuality with this group. Yet descriptions of these communication methods were largely absent in the primary research literature. Without more information about how adolescents with CP and complex communication needs currently communicate and want to communicate about sexuality, we will not recognize appropriate interventions or supports. To understand how people with complex communication needs communicate about sexuality, research must include them directly as participants rather than relying on proxy reports from parents or professionals. The work of Sellwood et al. exemplifies this inclusive approach.^50^

The literature described biological, psychological, and social factors that may affect communication about sexuality for adolescents with CP. Predominant biological factors included movement limitations and language comprehension difficulties. Psychologically, the available evidence predominantly addressed adolescents with CP who speak. This evidence outlined several considerations, including their priorities, the intersection of disability and sexual identities, self‐esteem, and internalized stigma. While scant, some findings also suggested that adolescents with CP may be trying to internally resolve tensions between their own desires and society's perceptions of their sexuality. This reflected findings from mainstream literature (identified in our framework search, step 1) showing that social norms do affect the way people imagine, expect, or plan interactions about sexuality.^28^, ^29^, ^42^, ^43^, ^44^, ^45^ The theory of planned behaviour^28^ may help to frame future research into the way that adolescents with CP plan communication about sexuality, thereby providing an understanding of the link between the psychological dimension and these interactions, as well as facilitating the identification of useful resources and supports.

Reports from adolescents with CP who speak emphasize the limited opportunities to communicate about sexuality with peers and sexual or romantic partners. Although the research did not report on interactions with peers or sexual or romantic partners for adolescents with complex communication needs, current evidence demonstrates that these young people are at even greater risk of social isolation and have more reduced social networks than adolescents with CP who speak.^5^, ^51^ Consequently, we hypothesize that they lack opportunities to communicate about sexuality, perhaps to a greater extent than those who can speak. We recommend that supporters of adolescents with CP and complex communication needs facilitate opportunities to communicate about a wider range of sexuality topics with a wider range of people, including discussing the positive aspects of sexuality.

Our analysis highlighted the interconnected relationships between the overarching categories of content, context, and influencing factors. Development of augmentative and alternative communication system vocabulary also illustrates this interplay. Researchers have worked with adults with complex communication needs to co‐design such vocabulary for adult roles^10^ and vocabulary for reporting sexual violence.^52^ We argue that the next phase for this research is developing interventions that facilitate opportunities for adolescents with complex communication needs to use that vocabulary when communicating with peers and sexual or romantic partners. Further, understanding social factors such as stigma and infantilization could support better vocabulary development and may result in vocabulary development that includes more positive aspects of sexuality. Effective supports and interventions cannot be developed if we do not understand these intersecting components as parts of a whole. Given this, in Table 3 we present specific recommendations arising from this review for (1) researchers and policy‐makers in CP and (2) people providing support to adolescents with CP and complex communication needs.

**TABLE 3:** Recommendations for stakeholders working with adolescents with CP.

Researchers and policy‐makers	Supporters (including clinicians, support workers, parents, and users of alternative and augmentative communication)
Include adolescents with complex communication needs in CP research.	Ask adolescents with CP and complex communication needs about their experiences, needs, and wants about communication about sexuality.
Seek to understand interactions about sexuality from the perspective of adolescents with CP and complex communication needs.	Facilitate opportunities for adolescents with CP and complex communication needs to communicate about sexuality with a wider range of people about a wider range of topics.
Involve research partners who have complex communication needs in every stage of the research process.	
Counter infantilization in research methodologies by researching positive aspects of sexuality, involving people with CP and complex communication needs in research design, and checking researcher assumptions.	Counter infantilization by recognizing adolescents with CP and complex communication needs are sexual and gendered beings.
Capture demographic data related to LGBTIQ identity of adolescents with CP.	Acknowledge that people with CP, including those with complex communication needs, can identify as LGBTIQ.

Abbreviations: CP, cerebral palsy; LGBTIQ, lesbian, gay, bisexual, transgender, intersex, or questioning.

In step 1 of this review, our systematic search of frameworks to understand communication about sexuality as a phenomenon identified two useful a priori frameworks: the bio‐psycho‐social model^29^, ^30^ and the theory of planned behaviour.^28^ For example, when applied during the deductive analysis of the primary evidence, the bio‐psycho‐social model highlighted how the interaction of biological, social, and psychological factors and processes affects how adolescents with CP and/or complex communication needs communicate about sexuality. This model also aligns with the World Health Organization's contextual definition of sexuality.^2^ Nonetheless, existing frameworks including the bio‐psycho‐social framework and theory of planned behaviour were limited in how well they handled factors specific to the adolescent developmental period (e.g. the importance of peers, parents, and educators as communication partners) or to disability (e.g. the sexual impacts of motor impairment; interactions with support workers) that were identified through inductive coding. At the same time, the absence of some deductive themes such as ‘digital interactions’ in the primary CP research indicates that the disability discipline has much to learn from mainstream sexuality research. In light of this mismatch, we call for the development of a comprehensive framework, encompassing key components of content, context, and influencing factors, that could be applied to any adolescent communication about sexuality. We suggest that engaging with a unifying theory as proposed could improve overall rigour, depth, and integration of sexuality research for adolescents with and without disability alike.

Participating in sexuality research benefits participants. For example, one participant in the research of East and Orchard expressed that the experience was positive and helped her understand her sexual identity.^36^ This perspective, along with most of the research in this review, comes from adolescents with CP who speak. Unfortunately, the current literature base on this topic largely excludes the one‐third of people with CP who have complex communication needs,^7^, ^8^, ^9^ the half who have an intellectual disability,^53^ and the nearly half who use mobility aids.^8^ Further, studies that sought information about adolescents with complex communication needs did so through proxy reporting, resulting in more information about their communication partners' (e.g. parents, carers, educators) experiences than proxy reporting about adolescents. Excluding participants with more complex disability from directly participating not only limits their opportunity to benefit from the research but also prevents us from understanding how different aspects of CP intersect to affect interactions about sexuality. We recommend that future sexuality research with people with CP directly involves participants with complex communication needs, intellectual disability, and those who use mobility aids, in line with recommendations for best practice.^54^, ^55^, ^56^ In a clinical or personal support setting, we recommend that supporters ask people with complex communication needs about their experiences, needs, and wants about communication about sexuality.

Despite the relative recency of the included studies, only two papers reported on sexual orientation,^14^, ^16^ and none reported on sex assigned at birth, gender identity, or innate variations of sex characteristics (also known as intersex). Sexual orientation and gender identity are central to the World Health Organization's definition of sexuality^2^ and to adolescent sexual development. Lesbian, gay, bisexual, transgender, intersex, or questioning (LGBTIQ) people with intellectual disabilities experience ‘dual marginalization’.^57^ We recommend that clinicians and other supporters acknowledge and support LGBTIQ identity development in adolescents with disabilities. This would be aided by research with people with CP, including those with complex communication needs, that better captures LGBTIQ demographic data, consistent with recent recommendations.^58^, ^59^

Research quality is improved when people with disabilities are included as participants, as well as when they drive the research as consumer research partners.^60^ Consumer involvement in this review process expanded the research team's understanding of the themes and links in the existing literature, highlighted key priorities towards useful, applicable results, and contributed to critical investigation of the gaps in the current literature. We paid particular attention to consumer involvement in our quality assessment (in QuADS, this related to stakeholder input). While four of the 16 papers reported some degree of stakeholder input (e.g. through the use of a pilot study^24^, ^33^, ^37^, ^38^), none reported sufficient stakeholder input for us to evaluate that involvement or understand the impact of that involvement (e.g. through the use of a tool such as the Guidance for Reporting Involvement of Patients and the Public 2^31^). We recommend that researchers involve research partners who have complex communication needs in every stage, and that involvement outcomes are measured and reported.

The recommendations from this study, summarized in Table 3, are specifically targeted for stakeholders working with adolescents with CP, differentiated for researchers and policy‐makers, and supporters (including clinicians and parents). We appreciate that these points may also be more broadly useful (e.g. with adults with CP, with other people with complex communication needs).

There are several limitations of this study. In line with the purpose of a scoping review, studies of any size were included. However, because of the small numbers of participants and limited number of primary research papers, findings from this review should be interpreted with caution, especially when generalizing the findings. Inconsistent terminology and classification of complex communication needs made it challenging to identify and extract data from, and report on, the papers that included (or excluded) people with complex communication needs. Beyond scale, some of the evidence was evaluated as low quality. We also acknowledge that reviewing grey literature is recommended in systematic reviews (including scoping reviews), in part because emerging health research and negative findings are more likely to be found in grey literature.^19^ Our review questions centred on what rigorous, empirical, peer‐reviewed evidence currently existed that could inform our understanding of communication about sexuality for adolescents with CP and complex communication needs, hence the exclusion of grey literature.

## CONCLUSION

This review illuminates a striking gap in the adolescent sexuality literature, where adolescents with CP and complex communication needs are profoundly underrepresented. From the evidence we found, it is clear that adolescents with CP want to communicate about sexuality more broadly, more often, and with a wider range of people. Further, it seems that adolescents with complex communication needs have even more limited opportunities for communication about sexuality than their peers with CP who speak. By mapping this gap and critically analysing how existing research constructs communication about sexuality for this population, we exposed urgent theoretical, methodological, and engagement challenges. Addressing these gaps is essential to realizing the rights of adolescents with disability to sexual health, agency, and inclusion.

Finally, exclusion from participation in research perpetuates the gaps in evidence for those with complex communication needs and denies them yet another opportunity to communicate about sexuality. Stakeholders working with adolescents with CP, including those with complex communication needs, should focus on understanding adolescents' experiences and priorities surrounding sexuality. Considering how content, context, and influencing factors intersect can lead to a deeper understanding of adolescents' experiences and more rigorous design of interventions and supports.

## FUNDING INFORMATION

The authors disclosed receipt of the following financial support for the project reported in this article: MW's PhD research and advisory group remuneration were funded by Centre of Research Excellence: CP‐Achieve funded by the National Health and Medical Research Council (Grant ID: APP1171758).

## CONFLICT OF INTEREST STATEMENT

The authors have stated that they had no interests that might be perceived as posing a conflict or bias.

## Supporting information


**Figure S1:** Framework Search (Step 1).

**Figure S2:** PRISMA flow chart depicting search for primary research studies (step 2).

**Table S1:** Guidance for Reporting Involvement of Patients and the Public 2‐Short Form.

## Data Availability

Data sharing not applicable to this article as no datasets were generated or analysed during the current study.
