# Supplementary material for: Communication about sexuality for adolescents with cerebral palsy and complex communication needs: A scoping review with framework synthesis
Source: Dev Med Child Neurol. 2025 Sep 10;68(1):49–63. doi: 10.1111/dmcn.16479 (PMC12683302; doi:10.1111/dmcn.16479)
Supplement: Supplementary file 1 — Figure S1: Framework Search (Step 1). [file DMCN-68-49-s002.docx]

**Figure S1: Framework Search (Step 1)**

Walsh M, Sawyer SM, Watson JM, O’Shea A, Cranko G, Pacheco CM, et al. Communication about sexuality for adolescents with cerebral palsy and complex communication needs: A scoping review with framework synthesis. Dev Med Child Neurol 2025. https://doi.org/10.1111/dmcn.16479

**Framework Search PRISMA flow chart^20^**

**Identification of studies via databases and registers**

Records removed *before screening*:

Duplicate records removed (n = 60)

Records identified from 9 EBSCO Databases and 1 Embase Database: 210

**Identification**

Records screened

(n = 150)

Records excluded

(n = 88)

Reports sought for retrieval

(n =62)

Reports not retrieved

(n = 0)

**Screening**

Reports excluded: 50

Centres abuse (n = 15)

Does not explain interactions (n = 12)

Not a model, theory or framework (n = 10)

Other reasons (n= 13)

Reports assessed for eligibility

(n =62)

Studies included in review

(n = 12)

**Included**

**Supporting Information: Framework Search (Step 1)**

Walsh M, Sawyer SM, Watson JM, O’Shea A, Cranko G, Pacheco CM, et al. Communication about sexuality for adolescents with cerebral palsy and complex communication needs: A scoping review with framework synthesis. Dev Med Child Neurol 2025. https://doi.org/10.1111/dmcn.16479

**Framework Search Included Articles – References**

Astle S, Toews M, Topham G, Vennum A. To talk or not to talk: an analysis of parents’ intentions to talk with children about different sexual topics using the theory of planned behavior [Internet]. Sex Res Social Policy. 2021[cited 2024 Jun 25];19:705-21. Available from: https://link.springer.com/article/10.1007/s13178-021-00587-6

Coduto KD, Vendemia MA, Viverette S, Williams E. Exploring sexual, romantic, and platonic features and functions of the imagined interactions framework [Internet]. Imagin Cogn Pers. 2022[cited 2024 Jun 25];42:24-41. Available from: <https://doi.org/10.1177/02762366221089279>

Döring N, Krämer N, Mikhailova V, Brand M, Krüger TH, Vowe G. Sexual interaction in digital contexts and its implications for sexual health: A conceptual analysis [Internet]. Front Psychol. 2021[cited 2024 Jun 25];12. Available from: <https://www.ncbi.nlm.nih.gov/pubmed/34916999>

Gowen CW, Britt TW. The interactive effects of homosexual speech and sexual orientation on the stigmatization of men [Internet]. J Lang Soc Psychol. 2016[cited 2024 Jun 25];25:437-56. Available from: <https://doi.org/10.1177/0261927X06292769>

Gustavsson L, Johnsson JI, Uller T. Mixed support for sexual selection theories of mate preferences in the swedish population [Internet]. Evolutionary Psychology. 2008[cited 2024 Jun 25];6:575-85. Available from: <https://doi.org/10.1177/147470490800600404>

Henningsen DD, Henningsen ML, Valde KS. Gender differences in perceptions of women’s sexual interest during cross-sex interactions: an application and extension of cognitive valence theory [Internet]. Sex Roles. 2006[cited 2024 Jun 25];54:821-29. Available from: <https://link.springer.com/article/10.1007/s11199-006-9050-y>

Krahe B, Bieneck S, Scheinberger-Olwig R. Adolescents' sexual scripts: schematic representations of consensual and nonconsensual heterosexual interactions [Internet]. J Sex Res. 2007[cited 2024 Jun 25];44:316-27. Available from: <https://www.ncbi.nlm.nih.gov/pubmed/18321011>

Levin RJ. Facets of female behaviour supporting the social script model of human sexuality [Internet]. J Sex Res. 1975[cited 2024 Jun 25];11:348-52. Available from: <https://doi.org/10.1080/00224497509550912>

Owen A, Arnold K, Friedman C, Sandman L. Nominal group technique: an accessible and interactive method for conceptualizing the sexual self-advocacy of adults with intellectual and developmental disabilities [Internet]. Qual Soc Work. 2015[cited 2024 Jun 25];15:175-89. Available from: <https://doi.org/10.1177/1473325015589803>

Prekatsounaki S, Gijs L, Enzlin P. Dyadic sexual desire in romantic relationships: the dyadic interactions affecting dyadic sexual desire model [Internet]. Arch Sex Behav. 2022[cited 2024 Jun 25];51:417-40. Available from: <https://www.ncbi.nlm.nih.gov/pubmed/35031906>

Wilson NJ, Plummer D. Towards supporting a healthy masculine sexuality: Utilising mainstream male health policy and masculinity theory [Internet]. J Intellect Dev Disabil. 2014[cited 2024 Jun 25];39:132-36. Available from: <https://doi.org/10.3109/13668250.2014.894500>

Wilson NJ, Shuttleworth R, Stancliffe R, Parmenter T. Masculinity theory in applied research with men and boys with intellectual disability [Internet]. Intellect Dev Disabil. 2012[cited 2024 June 25];50:261-72. Available from: <https://www.ncbi.nlm.nih.gov/pubmed/22731975>
