# Supplementary material for: Communication about sexuality for adolescents with cerebral palsy and complex communication needs: A scoping review with framework synthesis
Source: Dev Med Child Neurol. 2025 Sep 10;68(1):49–63. doi: 10.1111/dmcn.16479 (PMC12683302; doi:10.1111/dmcn.16479)
Supplement: Supplementary file 2 — Figure S2: PRISMA flow chart depicting search for primary research studies (step 2). [file DMCN-68-49-s003.docx]

**Figure S2:** PRISMA flow chart depicting search for primary research studies (step 2).

Walsh M, Sawyer SM, Watson JM, O’Shea A, Cranko G, Pacheco CM, et al. Communication about sexuality for adolescents with cerebral palsy and complex communication needs: A scoping review with framework synthesis. Dev Med Child Neurol 2025. https://doi.org/10.1111/dmcn.16479

**Identification of primary research studies via databases and registers**

Records removed *before screening*:

Duplicate records removed

(n = 572)

Records marked as ineligible by automation tools (n = 0)

Records removed for other reasons (n = 0)

Records identified from 9 EBSCO Databases and 1 Embase Database: 6722

**Identification**

Records screened

(n = 6150)

Records excluded

(n = 5417)

Reports sought for retrieval

(n = 730)

Reports not retrieved

(n = 0)

**Screening**

Reports excluded: 714

Unable to distinguish diagnostic group (n = 185)

Not experiences of target group (n = 149)

Not empirical (n = 101)

Insufficient length (n=80)

Other reasons (n= 199)

Reports assessed for eligibility

(n = 730)

Studies included in review

(n = 9)

Reports of included studies

(n = 16)

**Included**

*From:*  Page MJ, McKenzie JE, Bossuyt PM, Boutron I, Hoffmann TC, Mulrow CD, et al. The PRISMA 2020 statement: an updated guideline for reporting systematic reviews. BMJ 2021;372:n71. doi: 10.1136/bmj.n71

For more information, visit: <http://www.prisma-statement.org/>
