# Supplementary material for: Communication about sexuality for adolescents with cerebral palsy and complex communication needs: A scoping review with framework synthesis
Source: Dev Med Child Neurol. 2025 Sep 10;68(1):49–63. doi: 10.1111/dmcn.16479 (PMC12683302; doi:10.1111/dmcn.16479)
Supplement: Supplementary file 3 — Table S1: Guidance for Reporting Involvement of Patients and the Public 2‐Short Form. [file DMCN-68-49-s001.docx]

**Table S1: GRIPP2-SF**

Walsh M, Sawyer SM, Watson JM, O’Shea A, Cranko G, Pacheco CM, et al. Communication about sexuality for adolescents with cerebral palsy and complex communication needs: A scoping review with framework synthesis. Dev Med Child Neurol 2025. https://doi.org/10.1111/dmcn.16479

| **Section and Topic** | **Item** | **Reporting** |
| --- | --- | --- |
| **Aim** | Report the aim of PPI in the study | The aims of PPI in this study were to:   - expand understanding of the themes and links in the literature, - critically investigate gaps in current literature, and - identify themes which are high priority for knowledge translation. |
| **Methods** | Provide a clear description of the methods used for PPI in the study | Methods used for PPI in this study included:   - research partner training on thematic analysis in the context of a scoping review and Carroll et al.’s protocol, including: - training on protocol and reasoning for protocol; - confirming mutual understanding of a-priori codes; - meeting as a group following individual thematic analysis of one article to review and revise; - subsequent thematic analysis discussed in one-on-one meetings with author MW; - iterative review of codes including inductive development of new codes; - meeting to discuss potential patterns, links and gaps; and - meetings to discuss overall analysis; - research partner education on what is involved in academic co-authorship; - consultation with additional advisory group member on framing of results and discussion in a way that is relevant and respectful to people with CP; - reporting back to advisory group to check overall findings were relevant, aligned with community priorities, and were presented in a respectful way; and - collaborative completion of GRIPP2-SF including reflections on personal and project-level outcomes. |
| **Results** | Outcomes—Report the results of PPI in the study, including both positive and negative outcomes | - Overall outcome: Increased depth of understanding of the literature, including connections in themes that MW hadn't seen and providing practical grounding for the evidence. GC wrote "I felt like our discussions about how and why we coded articles the way we did help (sic) to clarify and refine the most fundamental ideas." - Negative outcomes: Both found it emotionally difficult to read about the violations of basic human rights and denial of agency described in some of the papers. Time consuming for AAC user. - Positive outcomes: Research partners reported personal benefits including research skill development and better understanding of the issues facing adolescents with CP and complex communication needs (including practical considerations for their own lives). |
| **Discussion** | Outcomes—Comment on the extent to which PPI influenced the study overall. Describe positive and negative effects | - PPI was critical to the mapping and synthesis of evidence as well as the development of discussion in this literature review. - Ideally, there would have been more research partner involvement in the review, such as in search term selection. We were limited by funding. |
| **Reflections** | Comment critically on the study, reflecting on the things that went well and those that did not, so others can learn from this experience | - When people with CP and/or complex communication needs are involved as research partners, they will likely require more time to complete tasks and engage in conversations. Project budgets must account for that time and funding. - Research partner wellbeing and self-care should be discussed before embarking on a literature review, so that research partners are well prepared for any literature that is shocking or upsetting. Because wellbeing and self-care had already been discussed with research partners and we had previously established trusting relationships, GC and ChP were able to talk about their emotional responses to the papers and opt in or out of any potentially distressing content. They chose to engage with that content because it was important to them that themes like infantalisation, stigma, and lack of awareness of sexual needs was reported on. |
